# Gut microbiome and nutrition-related predictors of response to immunotherapy in cancer: making sense of the puzzle

**DOI:** 10.1038/s44276-023-00008-8

**Published:** 2023-08-02

**Authors:** Cecilia Hes, R. Thomas Jagoe

**Affiliations:** 1 Peter Brojde Lung Cancer Centre, Segal Cancer Center, Jewish General Hospital, Montreal, QC H3T 1E2 Canada; 2 Division of Experimental Medicine, Faculty of Medicine and Health Sciences, McGill University, Montreal, QC H4A 3J1 Canada; 3 Research Center of the Centre Hospitalier de l’Université de Montréal (CRCHUM), Montréal, QC H2X 0A9 Canada

## Abstract

The gut microbiome is emerging as an important predictor of response to immune checkpoint inhibitor (ICI) therapy for patients with cancer. However, several nutrition-related patient characteristics, which are themselves associated with changes in gut microbiome, are also prognostic markers for ICI treatment response and survival. Thus, increased abundance of *Akkermansia muciniphila*, *Phascolarctobacterium, Bifidobacterium* and *Rothia* in stool are consistently associated with better response to ICI treatment. *A. muciniphila* is also more abundant in stool in patients with higher muscle mass, and muscle mass is a strong positive prognostic marker in cancer, including after ICI treatment. This review explores the complex inter-relations between the gut microbiome, diet and patient nutritional status and the correlations with response to ICI treatment. Different multivariate approaches, including archetypal analysis, are discussed to help identify the combinations of features which may select patients most likely to respond to ICI treatment.

## Introduction

Immune checkpoint inhibitor (ICI) treatment is a novel form of anticancer treatment which has revolutionised the management of many different cancers over recent years [1]. ICI treatment acts to counteract tumour evasion of immune surveillance and makes the tumour cells targets for killing and clearance by the host’s immune system [2, 3]. Predictive tumour biomarkers have been identified which indicate if a tumour is more likely to respond to ICI treatment [1]. These include the programmed death-ligand 1 (PD-L1) expression on cancer cells, tumour mutational burden, mismatch-repair deficiency/microsatellite instability [1] and absence of epidermal growth factor receptor mutations [4]. However, even when one or more of these biomarkers are used, the overall response rates range between 36 and 75% after >12 weeks of treatment with ICI across different types of cancer and lines of treatment [5]. Thus, better biomarkers are needed to improve selection of patients most likely to benefit from ICI.

The host immune system is central to the success of ICI treatment, and given the interactions between the gut microbiome and the host immune system, it is highly plausible that features of the gut microbiome may both modulate efficacy of immunotherapy [6, 7] and be potential predictive biomarkers of response. Several nutrition-related features, including body weight and composition have also been correlated with outcomes of ICI treatment. However, gut microbiome and nutritional status are often closely inter-related and, in the context of ICI treatment for cancer, it is unclear whether they offer independent prognostic information and how best to capture and combine these data to improve patient selection for ICI.

## Gut microbiome and the immune system

Immune surveillance protects against developing cancer, as shown by the increased incidence of malignancy in those with primary immune deficiency [8]. The host gut microbiome is important for the correct functioning of the immune system [6, 7] and germ-free mice have depletion of many immune cell lines (e.g., regulatory T-cells (Tregs)) and circulating cytokines, which are restored to normal levels after faecal microbiome transplant [6]. In addition, the presence of gut bacteria primes specific immune mechanisms required for the full effect of certain cytotoxic chemotherapies. Thus, the full therapeutic anticancer effects of cyclophosphamide are only achieved if the T-cell-mediated immune response induced by gut translocation of specific Gram-negative bacteria is also intact, and the anticancer effect of cyclophosphamide is blunted in antibiotic-treated or germ-free animals [9]. This evidence for a role for gut bacteria in determining effective immune-mediated cancer cell targeting and killing, is now further supported by several studies showing an association between increased abundance of certain gut bacteria and better response to ICI treatment [10–18].

## The gut microbiome and ICI cancer treatment response

**Fig. 1:**
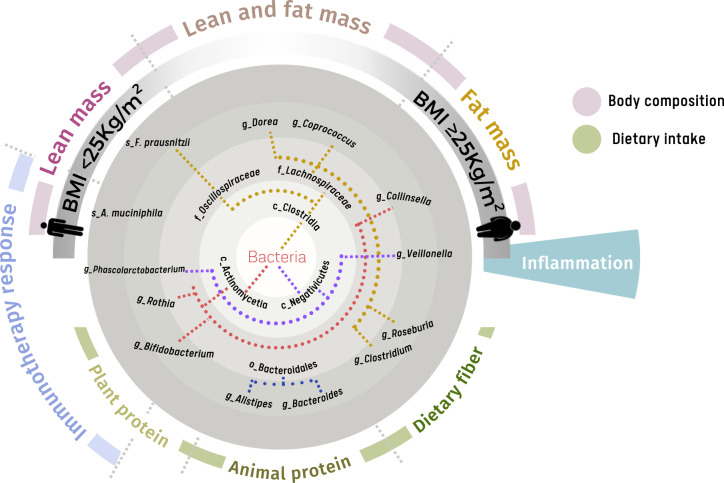
Gut microbiome bacteria consistently associated with markers of diet, nutritional status and ICI response. Bacterial taxa in stool that are associated with specific dietary components, markers of nutrition and body composition, and response to ICI cancer treatment. Selected bacterial taxa are shown that have been reported in at least two studies. Specific taxonomic levels are denoted by prefix: c_ class; f_ family; g_ genus; s_ species.

The most prominent taxa associated with good response to ICI (i.e., non-progressive disease) across different cancer types are *Phascolarctobacterium* [11, 14–16, 18, 19], *Bifidobacterium* [19–21], *A. muciniphila* [12, 13, 15, 16, 21, 22] and *Rothia* [11, 15, 16] (Fig. 1). In hepatobiliary cancers and metastatic melanoma, *Actinomyces* genus and members (e.g., *Actinomyces odontolyticus*) are consistently enriched in non-responders [10, 16, 17], whereas *Faecalibacterium prausnitzii* is increased in responders [10, 11, 14, 17]. In addition, across multiple advanced melanoma datasets *Roseburia* spp. associated with good response to ICI [19].

Oral antibiotics can disrupt the gut microbiome, and though it is not clear if the use of antibiotics alters abundance of all taxa identified as predictive of ICI response [18, 23], there is certainly evidence that antibiotic use close to the start of ICI treatment may impair treatment response [22, 24]. However, antibiotic-related changes in the gut microbiome do not affect outcomes of every type of ICI in the same way. In one study of melanoma patients treated with ICI targeting the cytotoxic T-lymphocyte antigen 4 (anti-CTLA-4), prior antibiotic use made no difference to outcomes, despite leading to a lower abundance of the *Bifidobacteriaceae* family [23]. In contrast, in another study decrease in the abundance of *Bifidobacterium* members with antibiotics use was associated with impaired response to ICI targeting the programmed cell death protein 1 (anti-PD-1) [25]. Overall, an effect of antibiotic use on ICI treatment outcomes is best described for patients with melanoma and lung and renal cancer treated with ICI combinations (i.e., anti-PD-1 plus anti-CTLA-4) or anti-PD-1 monotherapy. In these groups, contemporary antibiotic use is associated with lower response, i.e., shorter progression-free survival (PFS) [26]. It remains to be seen whether the association between the abundance of certain taxa in stool and ICI response is causative in humans and intervention trials using targeted faecal transplantation are ongoing (e.g., www.clinicaltrials.gov: NCT05251389) [27, 28].

## Cancer-bearing state and the gut microbiome

Taxonomic profiling of the gut microbiome has revealed that, compared to healthy subjects, *Veillonella* genus is usually more abundant in treatment-naïve patients with lung [29], pancreatic (*Veillonella atypica*) [30] and colorectal cancer [31, 32]. Furthermore, when comparing patients with advanced-stage cancer with and without weight loss, *Veillonella* is more abundant in those who lost weight [33]. Systemic inflammation is a common feature of advanced cancer and may contribute to metabolic changes promoting weight loss [34]. Consistent with this, *Veillonella* has also been found to be enriched in other inflammatory states such as autoimmune hepatitis and cystic fibrosis [35, 36].

Animal model studies have demonstrated that mice with acute leukaemia or colon cancer experiencing weight loss, have an increase in relative abundance of the *Enterobacteriaceae* family compared to controls [37]. Similar findings were found in patients with various cancer types and nutritional depletion [33]. By contrast, weight-losing animals with acute leukaemia or colon cancer had lower abundance of *Lactobacillus* spp. versus controls [37, 38]. However, there are discordant results for this taxon in depleted lung cancer patients versus healthy controls, with one study showing reduced *Lactobacillus* abundance [39] and another, reporting the opposite trend [33].

Studies of the microbiome in colorectal cancer have reported a more specific taxonomic pattern. In those patients the relative abundances of *Fusobacterium* [31, 32, 40, 41], *Porphyromonas* [31, 32, 40] and *A. muciniphila* [32, 42] are increased, and *F. prausnitzii* is decreased compared to healthy controls [32, 41]. Indeed, the enrichment of *Porphyromonadaceae* family [40], including *Porphyromonas somerae*, is reported in the presence of pre-malignant colonic lesions, suggesting a possible interaction between gut microbiome and the development of colorectal malignancy [31].

In general, the cancer-bearing state is associated with altered gut microbiome in animal models and in humans. The mechanisms underlying this observation are not fully understood, but as described below, there are many potential contributory factors which will vary in importance between individuals.

## Dietary intake and microbiome composition

Dietary intake is a major determinant of the human gut microbiome since bacteria obtain nutrients from the residue of the diet in the intestine [43]. The quantity and quality of the dietary residue present in the intestine has a differential influence on the growth and abundance of gut microbes and thus the composition of the gut microbiome [43, 44]. For example, a diet enriched in certain macronutrients (e.g., fat, protein) would favour the proliferation of species that are high metabolizers of those macronutrients [44, 45].

Both total fibre and specific fruit and vegetable fibre intake, are associated with the relative abundance of bacteria of the Clostridia class in the gut microbiome [46, 47], especially *Roseburia* genus [45, 48–51]. Many members of the *Clostridium* genus are also associated to higher dietary fibre intake [49, 52]. Differing dietary protein sources also favour prevalence of different bacteria. Thus, increased animal protein intake is associated with increased relative abundance of *Alistipes* [45, 50] and *Bacteroides* genera [45, 48, 50, 53], whereas plant protein intake correlates more closely with enrichment of some members of the *Bifidobacterium* genus (e.g., *B. longum*) [53, 54].

Food products are highly complex matrices [55], and their components as well as food preparation methods affect nutrient availability in the gut [56]. Moreover, nutrient–nutrient interactions may modulate nutrient uptake and net availability for gut bacteria [57]. There is evidence that a major change in diet can drive corresponding changes in that individual’s microbiome composition [50], but these changes do not persist when the new dietary intervention ceases [45]. Johnson et al. evaluated consumption of food products through food diaries in 34 subjects during 17 days with parallel analysis of each individual’s microbiome [58]. In that study, microbiome composition reflected dietary intake over a few days prior to sampling [58]. However, microbiome adaptations in response to a given combination of food products in one person did not predict changes seen in others in the same cohort [58]. Thus, individual host factors appear important in determining gut microbiome adaptations to diet. From a therapeutic standpoint, the extent to which dietary interventions alone can drive predictable changes in the gut microbiome remains uncertain.

## Cancer-related clinical factors that can modulate the gut microbiome

Patients with advanced cancer are often found to have severely altered and decreased dietary intake [34, 59]. There are many different causes for this but anorexia [59, 60] and taste and smell abnormalities related to cancer [61] and cancer treatment [62, 63] are common. Many cancer treatments, including ICI, can also cause other symptoms such as nausea or diarrhoea [63–66]. Severe disruption of nutrient intake is especially problematic for patients with upper gastrointestinal malignancies. Radiation-induced mucositis and dysphagia are common problems for head and neck cancer patients [67, 68] and can limit swallowing due to obstruction or pain [68]. Oesophageal and gastric cancers can also cause partial or complete obstruction [69]. The net result of the different barrier symptoms or physical changes, is that macronutrient intake and nutritional status declines [59, 62, 70] and affected patients are unable to maintain adequate dietary intake [59] and frequently lose weight [65, 67].

Not only is the amount and content of intestinal residue altered by the changes in dietary intake described, but the physiochemical environment in the bowel can also be affected. Bowel surgery, treatment-induced enteritis, atrophy or malabsorption [71] including bile-acid malabsorption [72] are common causes for persistent diarrhoea in patients with cancer. Such changes can alter luminal pH [73] and growth conditions [74] for gut bacteria. Taken together, the changes in food intake and the alterations in bowel function and luminal conditions can have a profound impact on the gut microbiome.

## Patient nutritional status and response to ICI treatment

Weight loss and low body weight are two of the most powerful negative prognostic indicators of outcome in cancer [75–78]. In general, patients at more advanced stages of disease suffer greater weight losses [79] and reduced dietary intake appears to be the strongest predictor of weight loss [34]. For the subgroup of patients treated with ICI, there have been mixed reports about the prognostic importance of weight loss. Some studies showed that in non-small cell lung cancer (NSCLC) patients, those with recent pre-treatment weight loss had shorter PFS [80–82] whilst other studies did not [83, 84]. Similarly, in patients with squamous cell head and neck carcinoma (HNSCC), weight (expressed as weight(kg)/height(m)^2^ or body mass index (BMI)) change over 4–7 months prior to ICI treatment was not a predictor of PFS [85], whereas another study in mixed cancer types, greater reduction in BMI over a shorter interval (15–45 days) prior to ICI treatment was a predictor of poor PFS [86]. Weight loss is also a predictor of worse overall response rates with ICI treatment [84, 86].

A number of studies have reported that overweight (BMI ≥25 kg/m^2^) or obesity (BMI ≥30 kg/m^2^) predicts improved overall survival (OS) rates after treatment with ICI in NSCLC [87, 88] and melanoma [89–91]. In NSCLC the relationship may be non-linear with less benefit for obese than in those who are only categorised as overweight [92]. However, the relationship between BMI and PFS after ICI treatment is less clear. Kichenadasse et al. found overweight and obese patients with NSCLC had improved PFS after treatment with atezolizumab but only in those with PDL-1-positive tumours (tumour proportion score >5%) [87]. In contrast, three recent studies including NSCLC patients treated with ICI showed that BMI is not a predictor of PFS [82, 83, 93].

The changes in body composition and decline in muscle and fat tissue mass in patients with advanced cancer are well described [94, 95] and the prognostic importance of these differences in body composition is now clear. Depletion of muscle mass has been shown to be an especially poor prognostic factor for cancer treatment outcomes [94] including ICI. In patients with lung cancer, reduced muscle mass is strongly associated with worse PFS after ICI use [96] with a >eightfold increased risk for progressive disease [97]. Furthermore, in refractory and metastatic HNSCC patients treated with nivolumab, higher values of skeletal muscle had better response rates and PFS [98]. This relationship has been confirmed in meta-analyses, including multiple different types of cancer [99–102].

Patients with greater subcutaneous fat stores are reported to have longer PFS in various cancer types treated with ICI [103, 104]. However, the type of adipose tissue and the corresponding status of other tissue stores may be important in determining any relationship between fat mass and response to ICI. In one cohort of NSCLC patients, visceral fat and visceral/subcutaneous fat ratio did not relate to PFS or response [105], whereas in another study of NSCLC, total adipose tissue (i.e., visceral plus subcutaneous fat) levels did correlate with better ICI response and PFS, but only in patients who were weight-stable at start of the treatment [84]. Similarly, patients with HNSCC, renal cell and urothelial carcinomas who had both lower visceral fat and skeletal muscle mass were at higher risk of progressive disease in most [98, 106, 107], but not all studies [104].

## Gut microbiome and nutritional status

Gut microbiome studies in humans show *F. prausnitzii* abundance is greater in subjects with lower BMI in comparison to subjects with obesity [108, 109]. In contrast, subjects with BMI values ≥25 kg/m^2^ have higher relative abundances of members of the *Collinsella* genus (e.g., *Collinsella aerofaciens*) [109, 110], and the *Veillonellaceae* [48, 111] and *Lachnospiraceae* families (e.g., genera *Dorea*, *Lachnospira*, *Coprococcus*) [52, 109, 110, 112–115] (Fig. 1).

Animal models suggest that the gut microbiome modulates muscle mass as evidenced by reversal of muscle atrophy and increase on protein synthesis observed in germ-free mice after faecal transplant [116]. In humans, higher muscle mass and a leaner body composition (i.e., relatively lower fat mass), is associated with enrichment of *Akkermansiaceae* family members in the gut microbiome [117, 118], including *A. muciniphila* [119]. *Coprococcus* genus and *Lachnospiraceae*, were more abundant in subjects with BMI ≥25 kg/m^2^ and those with greater skeletal muscle mass [120]. Abundance of *Faecalibacterium* genus members (i.e., *F. prausnitzii*) is greater in both women [118, 120] and men [51] with higher skeletal muscle mass (Fig. 1). Furthermore, increases in both lean mass and *Faecalibacterium* relative abundance are observed in normal weight subjects after exercise training [110]. This appears consistent with lower abundance of *F. prausnitzii*, as well as members of the Clostridiales class including *Eubacterium* and *Roseburia* genera, in older subjects with physical frailty and sarcopenia, compared to controls [121–123].

Studies focused on adipose tissue have shown *A. muciniphila* abundance is inversely correlated with total fat mass in animal models [124] and subcutaneous fat (rather than total body fat) in human studies [125]. On the other hand, *Coprococcus* abundance correlates positively with level of subcutaneous body fat [115]. Interestingly, there are some geographic differences in results. For example, in western populations, *Blautia* genus abundance is directly correlated with both BMI and visceral fat [113], but the inverse relationship is observed in studies in Japanese and Chinese populations where decreasing in relative abundance of *Blautia* in the gut microbiome, is associated with increased visceral fat [115, 126]. The reasons for these differences are not clear, but similar observations have been reported for *Bifidobacterium* and *Oscillospira* genera, with higher relative abundances in overweight and obese subjects or subjects with higher visceral fat values in Western countries [109, 113], but not in East and South-Asian populations [126–128].

In summary, certain taxa are enriched in subjects with higher relative fat or muscle mass. The mechanisms leading to these differences in gut bacterial abundance in subjects with different body weight and composition are unknown. However, given the association between higher muscle mass and favourable prognosis in cancer, body composition is another potentially important prognostic marker linking microbiome profile and response to ICI treatment.

## Multivariate analysis to predict response to ICI therapy in cancer

ICI treatment is now established as a highly effective form of cancer treatment. However, despite the use of validated tumour biomarkers, it remains challenging to select the patients who will achieve sustained response to ICI. Features of the gut microbiome and other patient-related nutritional factors have now been shown to predict response to ICI in lung cancer. It seems highly plausible that using a combination of microbiome and nutrition-related biomarkers, in addition to current tumour-based biomarkers, will provide more accurate predictions of ICI response.

**Fig. 2:**
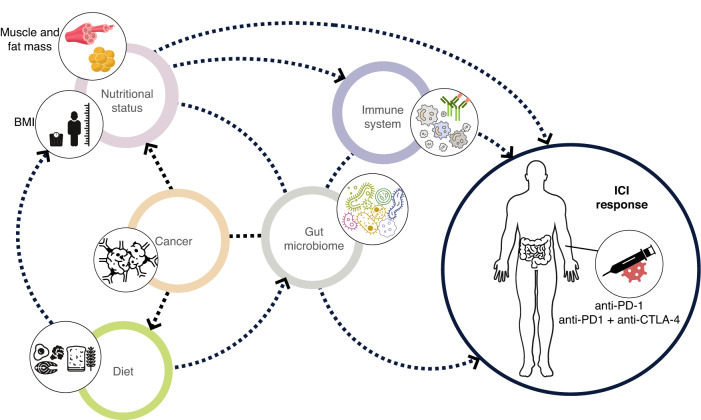
Inter-relationships between diet, nutritional status, gut microbiome and response to ICI in cancer. Diet is a major determinant of nutritional status. Dietary intake and nutritional status are associated with changes in the gut microbiome composition in healthy and diseased subjects. Changes in diet, nutritional status and the gut microbiome are often found in patients diagnosed with cancer. Immune function is modulated by the gut microbiome composition and nutritional status, and features of nutritional status and gut microbiome are associated with different tumour responses to ICI.

As outlined above, there is good evidence that the gut microbiome composition is influenced by diet which may also determine some features of nutritional status and body composition (Fig. 2). Thus, there may be confounding effects when trying to combine such features for determining prognosis with ICI treatment. Even the association between gut bacterial abundance and immune-mediated anticancer activity may be modified by nutritional status, as the association between severe protein-energy depletion and impaired immune function is well known [129, 130]. Hence, identifying those with depleted nutritional status may also highlight patients less likely to be able to mount the host anti-tumour immune response needed to get the benefits of ICI treatment. To date the inter-relationships between the gut microbiome and nutritional factors in determining outcomes of ICI treatments have not been fully explored.

Given the potential bi-directional relations between gut microbiome and patient nutritional characteristics, sophisticated multivariate analyses are needed to develop robust predictive biomarkers. Some recent studies have performed multivariate analysis combining clinical factors such as age, performance status, weight (BMI), weight loss and body composition, tumour biomarker expression (e.g., PD-L1 expression) and immune-related factors (e.g., cytokines and counts of circulating immune cells) to determine which features independently predict response to ICI in cancer [82, 83, 88, 92, 131]. However, to date, multivariate analyses to predict ICI treatment response have not incorporated gut microbiome data. Large datasets including microbiome data have only recently become more widely available and there are still challenges in standardising data processing and incorporating data of this type and structure into standard statistical modelling approaches.

**Fig. 3:**
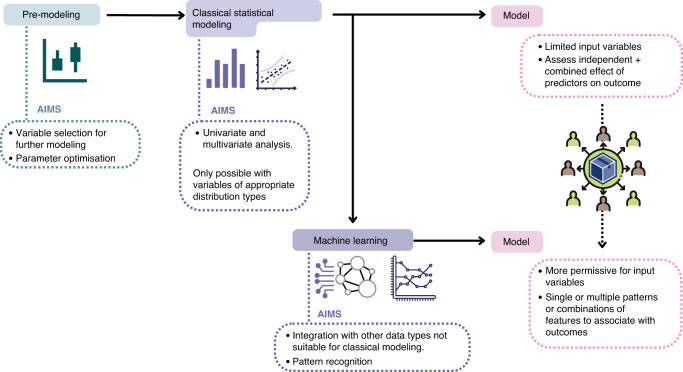
Modelling predictive biomarkers for ICI response: integrating multidimensional datasets. A schema is outlined to combine classical statistical modelling and machine-learning pattern-recognition approaches to predict outcomes of ICI treatment. Statistical modelling can determine the best single set of explanatory markers which predict outcomes. However, there are restrictions on the types of data which can be modelled using this approach alone and, when including nutritional and microbiome data, the assumption that there is only a single best set of explanatory variables may be flawed. Combining results of statistical modelling with machine-learning approaches allows the inclusion of data not easily analysed using classical modelling and identification of key patterns within highly inter-related multidimensional data types. This approach can be used to analyse clinical and molecular data to identify different combinations of features which predict response and outcomes from ICI treatment in cancer.

To address this, one solution to modelling is to combine statistical analysis (e.g., regression) and machine-learning algorithms to find the best predictive biomarkers [132–134]. For instance, a statistical-based selection of variables to include in a learning model can be done as a pre-modelling step (Fig. 3) [133, 134]. However, rather than relying on classical statistical models alone, other data, such as high-throughput sequence data including relative abundance of selected bacteria taxa in each individual, can then be combined with variables selected in the pre-modelling step and analysed using machine-learning approaches. A pattern-recognition approach has been proposed by other authors to harness the potential of high-throughput data to predict prognosis in cancer [135] and achieve optimal personalised cancer treatment.

One promising method is archetypal analysis, in which the values of biomarkers in all individuals is used to generate a number of ‘archetypes’ (i.e., pure/extreme patterns that explain all patterns across multiple variables observed in a sample) [136]. Individual subjects can then be matched to one or more archetype and the confidence of assignment to a given archetype can be calculated. The results of the archetypal analysis are easily interpreted [137] and suitable for use with data such as gut microbiome data that cannot be analysed using regression modelling and classic multivariate analysis [138]. Archetypal analysis has been successfully applied to find combinations of laboratory and clinical features predicting trajectories of chronic infection in cystic fibrosis patients [139] and for prediction of allograft survival in renal transplant recipients [140]. More recently, we have used this approach in conjunction with statistical modelling in a cohort of patients with NSCLC, to determine the combinations of nutritional, clinical and microbiome data associated with response to ICI treatment (unpublished). The results revealed a limited number of archetypes, two of which were associated with better PFS. The two favourable archetype patterns had distinct body composition profiles (e.g., high vs low body fat) and patterns of dietary intake (e.g., high vs low fibre intake) and differing gut relative abundance of the selected taxa included in the analysis.

Importantly, when studying biomarkers which are potentially highly inter-related such as for the gut microbiome and markers of diet and nutrition in response to ICI treatment, it is plausible that the impact of alterations in one factor (e.g., body fat), may be mitigated by changes in other related factors such as gut microbiome. Thus, as demonstrated by our own findings mentioned above, multiple different combinations of these factors may exist which are associated with better or worse outcomes. Archetypal analysis and similar approaches are useful tools to try and identify these different combinations of factors . This in turn raises questions about whether the archetypes identified can help understand the nutritional and microbiome-related mechanisms promoting better response to ICI and eventually inform intervention studies to improve outcomes.

## Conclusions

Despite the use of current tumour-based biomarkers it remains challenging to predict whether a given individual will have sustained response to ICI treatment. The patient’s gut microbiome profile and many nutrition-related features provide additional prognostic information, but it is not clear how best to incorporate these data to improve ICI treatment planning. A combination of statistical modelling and machine-learning techniques, such as archetypal analysis, is proposed to identify patterns or combinations of features associated with better outcomes after ICI treatment. This pattern-recognition approach may also identify mechanistically important co-dependencies between nutritional and gut microbiome data and lay the groundwork for future intervention studies to improve outcomes.

## Data Availability

Not applicable.
